# Radionuclide Molecular Imaging of EpCAM Expression in Triple-Negative Breast Cancer Using the Scaffold Protein DARPin Ec1

**DOI:** 10.3390/molecules25204719

**Published:** 2020-10-14

**Authors:** Anzhelika Vorobyeva, Ekaterina Bezverkhniaia, Elena Konovalova, Alexey Schulga, Javad Garousi, Olga Vorontsova, Ayman Abouzayed, Anna Orlova, Sergey Deyev, Vladimir Tolmachev

**Affiliations:** 1 Department of Immunology, Genetics and Pathology, Uppsala University, 751 85 Uppsala, Sweden; javad.garousi@igp.uu.se (J.G.); olga.vorontsova@igp.uu.se (O.V.); vladimir.tolmachev@igp.uu.se (V.T.); 2 Research Centrum for Oncotheranostics, Research School of Chemistry and Applied Biomedical Sciences, National Research Tomsk Polytechnic University, 634 050 Tomsk, Russia; yekaterinabezv@mail.ru (E.B.); schulga@gmail.com (A.S.); anna.orlova@ilk.uu.se (A.O.); biomem@mail.ru (S.D.); 3 Department of Pharmaceutical Analysis, Siberian State Medical University, 634050 Tomsk, Russia; 4 Molecular Immunology Laboratory, Shemyakin & Ovchinnikov Institute of Bioorganic Chemistry, Russian Academy of Sciences, 117997 Moscow, Russia; elena.ko.mail@gmail.com; 5 Department of Medicinal Chemistry, Uppsala University, 751 23 Uppsala, Sweden; ayman.abouzayed@ilk.uu.se; 6 Science for Life Laboratory, Uppsala University, 751 23 Uppsala, Sweden; 7 Bio-Nanophotonic Lab, Institute of Engineering Physics for Biomedicine (PhysBio), National Research Nuclear University ‘MEPhI’, 115409 Moscow, Russia; 8 Center of Biomedical Engineering, Sechenov University, 119991 Moscow, Russia

**Keywords:** EpCAM, radionuclide, molecular imaging, SPECT, iodine, PIB, breast, cancer

## Abstract

Efficient treatment of disseminated triple-negative breast cancer (TNBC) remains an unmet clinical need. The epithelial cell adhesion molecule (EpCAM) is often overexpressed on the surface of TNBC cells, which makes EpCAM a potential therapeutic target. Radionuclide molecular imaging of EpCAM expression might permit selection of patients for EpCAM-targeting therapies. In this study, we evaluated a scaffold protein, designed ankyrin repeat protein (DARPin) Ec1, for imaging of EpCAM in TNBC. DARPin Ec1 was labeled with a non-residualizing [^125^I]I-*para*-iodobenzoate (PIB) label and a residualizing [^99m^Tc]Tc(CO)_3_ label. Both imaging probes retained high binding specificity and affinity to EpCAM-expressing MDA-MB-468 TNBC cells after labeling. Internalization studies showed that Ec1 was retained on the surface of MDA-MB-468 cells to a high degree up to 24 h. Biodistribution in Balb/c nu/nu mice bearing MDA-MB-468 xenografts demonstrated specific uptake of both [^125^I]I-PIB-Ec1 and [^99m^Tc]Tc(CO)_3_-Ec1 in TNBC tumors. [^125^I]I-PIB-Ec1 had appreciably lower uptake in normal organs compared with [^99m^Tc]Tc(CO)_3_-Ec1, which resulted in significantly (*p* < 0.05) higher tumor-to-organ ratios. The biodistribution data were confirmed by micro-Single-Photon Emission Computed Tomography/Computed Tomography (microSPECT/CT) imaging. In conclusion, an indirectly radioiodinated Ec1 is the preferable probe for imaging of EpCAM in TNBC.

## 1. Introduction

Breast cancer is one of the most common types of cancer among women worldwide. It is a heterogeneous disease, which is categorized into four subtypes: Luminal A, luminal B, human epidermal growth factor receptor 2-positive (HER2-positive), and “basal-like” or “triple-negative” [1]. Triple-negative breast cancer (TNBC) does not express estrogen, progesterone, or human epidermal growth factor (HER2) receptors and is characterized by having an aggressive course, early metastatic spread, and poor prognosis [2]. Although initial response to chemotherapy in TNBC might be better compared to other breast cancer subtypes, early relapse is commonly observed [3,4,5]. Furthermore, the high rate of relapse among TNBC patients after surgery because of incomplete eradication of the tumor highlights the need for more effective therapies [2].

Targeted systemic treatment is well proven for estrogen/progesterone- or HER2-expressing breast cancer but is still not established to the same extent for TNBC [1]. Therefore, disseminated TNBC is particularly challenging to treat. The standard of care for TNBC patients is sequential chemotherapy. However, its indiscriminate toxicity to healthy tissues results in a narrow therapeutic window and limited efficacy. Targeted delivery of a cytotoxic payload (drug, toxin, or radionuclide) specifically to cancer cells would reduce the off-target toxicity and increase the therapeutic window and the efficiency of the treatment. In April 2020 the US Food and Drug Administration (FDA) approved the antibody-drug conjugate sacituzumab govitecan-hziy, which targets the tumor-associated calcium signal transducer 2 (TROP-2) antigen, for treatment of disseminated TNBC refractory to previous chemotherapies [6]. It showed a 33% response rate in patients with metastatic TNBC pretreated with chemotherapy [7]. Still, the development of therapeutics specific to other molecular targets could further increase the success rate in treatment of TNBC.

The epithelial cell adhesion molecule (EpCAM) is overexpressed in a large number of TNBC cases. The fraction of EpCAM overexpression in TNBC is 36–88%, depending on the scoring system [8,9,10], which makes it an attractive target for this malignancy. In patients with TNBC, EpCAM overexpression is associated with unfavorable prognosis [9] and correlates with poor survival, lymph node metastasis, and distant metastasis [11]. Several therapeutic strategies targeting EpCAM in TNBC are under preclinical and clinical development [12,13,14]. The anti-EpCAM monoclonal antibody adecatumumab has been evaluated in a phase II clinical study in patients with metastatic breast cancer [15]. Three of the 18 patients with high EpCAM expression and adecatumumab treatment developed new metastases up to week 6, compared with 14 of 29 patients with low EpCAM expression, indicating that response was related to EpCAM expression.

Due to heterogeneity of EpCAM expression in patients with TNBC, it is necessary to select the patients with high expression for EpCAM-targeted therapy. Radionuclide molecular imaging allows for non-invasive, whole-body evaluation of targeted protein expression. In the past years, a number of biomolecules against various targets have been investigated for radionuclide molecular imaging of breast cancer, such as somatostatin (SST) analogues targeting SST receptors, vasoactive intestinal peptide targeting affibody molecules against HER2, arginine-glycine-aspartate (RGD) peptides targeting integrin receptors, bombesin analogues targeting gastrin-releasing peptide receptors (GRPRs), and peptide analogues of alpha-melanocyte stimulating hormone-targeting melanocortin receptors [16].

Previously developed probes for radionuclide molecular imaging of EpCAM were mainly based on a monoclonal antibody (mAb) scaffold [17,18,19]. In comparison to mAbs, engineered scaffold proteins (ESP) have more favorable properties for imaging because of their small size, which enables rapid localization in tumors and fast decrease of blood-associated background activity due to renal clearance [20,21]. Additionally, ESPs can be genetically engineered to incorporate peptide-based chelators, e.g., histidine tags for labeling with technetium-99m tricarbonyl, and can generally tolerate harsh radiolabeling conditions, such as high temperature or changes in pH. A class of ESP, the designed ankyrin repeat proteins (DARPins), demonstrated excellent results for radionuclide molecular imaging of HER2 in preclinical studies, providing high tumor-to-nontumor tissue ratio shortly after injection [22,23,24]. The DARPin scaffold consists of four to six helix-turn-helix units and has molecular weight from 14 to 18 kDa, depending on the number of units. DARPins are currently the only class of ESPs with binders selected against EpCAM [25]. According to surface plasmon resonance, DARPin Ec1 has affinity of 68 pM to EpCAM [25], which meets requirements for a high-affinity imaging probe.

High affinity of an imaging probe is an important precondition for successful imaging, but it is not sufficient. Selection of a radionuclide and chemistry for its conjugation to a targeting probe is essential. Experience with other targeting proteins (affibody molecules [26] and DARPins targeting HER2) [22,23,24,27] demonstrated that the selection of an optimal labeling approach can increase tumor-to-organ ratios by an order of magnitude. Modification of the protein surface by a radionuclide in combination with a chelator or linker for coupling results in the alteration of off-target interactions with blood vessels and normal tissues. This has an essential impact on unspecific uptake in normal organs and tissues. Furthermore, accumulation of activity in both tumors and normal tissues depends on physicochemical properties of radioactive metabolites, which are formed after internalization and intracellular proteolysis of a labeled protein. Labels having charged or bulky polar radiometabolites are trapped inside the cells after proteolysis. Such labels are called *residualizing* [28]. The residualizing labels provide a long retention of activity both in tumors and in normal tissues if the imaging probe is internalized. In the case when the radiometabolites are lipophilic, they are capable of diffusing through cellular membranes and leaving the cell. These so-called *non-residualizing* labels are associated with low cellular retention of activity after internalization. When the internalization of an imaging probe by malignant cells is rapid, the use of residualizing labels is the only option for sufficient accumulation of activity in a tumor. However, the use of non-residualizing labels might be appropriate when internalization of a targeting probe by the cancer cells is slow, and an unspecific uptake, first and foremost, in liver and kidneys results in rapid internalization in normal tissues. In this case, tumor retention of activity depends mainly on the high affinity of a probe to its molecular target. A successful use of non-residualizing radiohalogen labels was demonstrated earlier for ESPs such as HER2-binding Albumin-binding domain (ABD)-Derived Affinity ProTein 6 (ADAPT6) [29] and HER2-binding DARPins 9_29 and G3 [22,23], as well as for Ec1 for imaging of EpCAM expression in pancreatic and ovarian cancer models [30,31]. Importantly, good retention in tumors was accompanied by a rapid clearance from normal tissues, which increased tumor-to-organ ratios. Apparently, slow internalization of Ec1 after binding to TNBC cells is critical for the use of a non-residualizing label for imaging in this cancer. Such a slow internalization of radiolabeled Ec1 was observed in the case of binding to pancreatic and ovarian cell lines [30,31], which was an indication that the cellular processing pattern would be similar for TNBC as well. However, our previous observations with ESP suggest that an internalization rate might depend on origin of cancer cells. For example, a rate of HER2-binding affibody [^111^In]In-DOTA-ZHER2:342-pep2 internalization by ovarian cancer cells was twice higher compared with a rate of internalization by breast cancer cells [32]. HER3-binding affibody-ABD fusion proteins were internalized by pancreatic cancer cells appreciably rapider than by prostate cancer cells [33]. Thus, evaluation of internalization of radiolabeled Ec1 by TNBC cells was necessary before in vivo experiments.

The goal of this study was to investigate whether our previous findings could be translated to triple-negative breast cancer and to evaluate the potential of DARPin Ec1 for imaging of EpCAM in a TNBC model in vivo. To evaluate the influence of residualizing properties of the radiolabel, technetium-99m tricarbonyl [^99m^Tc]Tc(CO)_3_ with residualizing properties was used as a comparator to the non-residualizing [^125^I]I-*para*-iodobenzoate ([^125^I]I-PIB) label. The ^99m^Tc (T_1/2_ = 6.01 h) is the most commonly used radionuclide in clinical single-photon emission computed tomography (SPECT). The ^125^I (T_1/2_ = 59.4 d) is a chemical analogue and a convenient preclinical surrogate for ^123^I (T_1/2_ = 13.27 h), which is used for SPECT, or ^124^I (T_1/2_ = 4.18 d), which is used for positron emission tomography (PET).

## 2. Materials and Methods

### 2.1. General Procedures

Sodium iodide [^125^I]NaI was purchased from PerkinElmer Sverige AB (Upplands Väsby, Sweden). Technetium-99m was obtained as pertechnetate by elution of Ultra-TechneKow generator (Mallinckrodt, Petten, The Netherlands) with sterile 0.9% sodium chloride (Mallinckrodt, Petten, The Netherlands). The CRS (Center for Radiopharmaceutical Sciences) kits for production of tricarbonyl technetium were purchased from the Center for Radiopharmaceutical Sciences (PSI, Villigen, Switzerland; contact e-mail: crs-kit@psi.ch). Instant thin-layer chromatography (iTLC) analysis was performed using iTLC silica gel strips (Varian, Lake Forest, CA, USA). The radioactivity distribution along iTLC strips was measured using a Cyclone storage phosphor system (Packard) and analyzed by OptiQuant image analysis software. Purification of radiolabeled proteins was performed using NAP-5 size-exclusion columns (GE Healthcare, Buckinghamshire, UK). Radioactivity was measured using an automated gamma-spectrometer with a NaI (TI) detector (1480 Wizard, Wallac, Finland). MDA-MB-468 breast cancer cells and Ramos lymphoma cells were purchased from the American Type Culture Collection (ATCC) and were cultured in Roswell Park Memorial Institute (RPMI) medium supplemented with 10% fetal bovine serum (FBS), 2 mM L-glutamine, 100 IU/mL penicillin, and 100 µg/mL streptomycin, in a humidified incubator with 5% CO_2_ at 37 °C, unless mentioned otherwise. Binding specificity and cellular processing experiments were performed using 35-mm Petri dishes (Nunclon Delta Surface, ThermoFisher Scientific, Roskilde, Denmark). Ligand Tracer experiments were performed using 89-mm Petri dishes (Nunclon Delta Surface, ThermoFisher Scientific, Roskilde, Denmark).

### 2.2. Protein Production and Radiolabeling

The EpCAM-targeting DARPin Ec1-H_6_ (containing a hexahistidine tag at C-terminus) was produced based on sequences published previously [25]. Production and purification of DARPin Ec1 was performed as described previously [30].

Indirect radioiodination of Ec1 using N-succinimidyl-para-(trimethylstannyl)benzoate was performed as described earlier [28,31]. Acetic acid in water (0.1%, 10 µL) was added to radioiodine (5–15 µL, 17–42 MBq). Then, N-succinimidyl-p-(trimethylstannyl)benzoate (13 nmoles, 5 µg, 5 µL of 1 mg/mL in 5% acetic acid in methanol) and chloramine-T (40 µg, 10 µL, 4 mg/mL in water) were added. The reaction was stopped by addition of sodium metabisulfite (60 µg, 10 µL, 6 mg/mL in water) after 5 min of incubation at room temperature. Then, DARPin Ec1 (7.6 nmoles, 140 µg, 39 µL of 3.6 mg/mL in phosphate-buffered saline (PBS)) in 140 µL of 0.07 M borate buffer (pH 9.3) was added and incubated at room temperature for 30 min. The radiolabeled conjugate was purified on a NAP-5 column, pre-equilibrated with 1% bovine serum albumin (BSA) in PBS, and eluted with PBS. The labeling yield and purity were determined using radio-iTLC analysis in 4:1 acetone:water system.

Site-specific radiolabeling of DARPin Ec1-H_6_ bearing a C-terminal His_6_-tag with tricarbonyl technetium-99m was performed as described earlier [31]. The solution eluted from the technetium generator (500 µL) containing 3–4 GBq of [^99m^Tc]Tc pertechnetate was added to the CRS kit, and the mixture was incubated at 100 °C for 30 min. The obtained solution of [^99m^Tc]Tc(CO)_3_ (12 µL, 83–108 MBq) was mixed with a solution of DARPin Ec1 (40 µg, 2.18 nmol) in 33 µL of PBS and incubated at 60 °C for 60 min. The radiochemical yield and purity were determined using iTLC strips eluted with PBS. The radiolabeled DARPin Ec1 was purified using NAP-5 columns pre-equilibrated and eluted with PBS.

Radio high-performance liquid chromatography (HPLC) analysis was performed using a Hitachi Chromaster HPLC system with a radioactivity detector and Phenomenex Luna^®^ C18 column (100 Å; 150 × 4.6 mm; 5 μm) at room temperature (20 °C). Solvent A was 0.1% trifluoroacetic acid (TFA) in H_2_O, solvent B was 0.1% TFA in acetonitrile, and the flow rate was 1 mL/min. For identity and purity analysis, the 20-min method with a gradient from 5 to 95% solvent B over 18 min and from 95% to 5% solvent B from 18 to 20 min was used.

The label stability under challenge conditions (excess of histidine for [^9 9m^Tc]Tc(CO)_3_-Ec1, NaI, or in 30% ethanol for [^125^I]I-PIB-Ec1) was assessed by analysis with iTLC silica gel (SG) strips eluted by PBS or by 4:1 acetone:water. The stability in complete cell culture medium containing 10% fetal bovine serum after 24 h of incubation at 37 °C was assessed by passing the media through a NAP-5 size-exclusion column and collecting the high-molecular-weight fraction (containing molecules over 5 kDa) and the low-molecular-weight fraction (containing molecules below 5 kDa). The activity in the column, the high- and low-molecular-weight fractions, was measured using a gamma-spectrometer.

### 2.3. Binding Specificity and Cellular Processing Assays

In vitro studies were performed using EpCAM-expressing breast cancer cell line MDA-MB-468. One day before the experiment, cells were seeded in 3-cm Petri dishes (ca. 1 × 10^6^ cells per dish) and three dishes per group were used.

Binding specificity to EpCAM was evaluated as described previously [30,31]. To saturate EpCAM receptors, 100-fold excess of nonlabeled Ec1 DARPin (200 nM) in cell culture medium was added to one group of cells and an equal volume of media only was added to the second group. After 30 min of incubation at room temperature, radiolabeled DARPins [^125^I]I-PIB-Ec1 or [^99m^Tc]Tc(CO)_3_-Ec1 were added at 2 nM final concentration. After 6 h of incubation at room temperature, the medium was collected, cells were washed, and trypsin was added to detach the cells. The cell suspension was collected, and the radioactivity of cells and medium was measured to calculate the percent of cell-bound radioactivity. The data were analyzed using unpaired two-tailed *t*-test.

Cellular retention and processing were studied during continuous incubation using an acid-wash method [32]. To study cellular processing during continuous incubation, radiolabeled [^99m^Tc]Tc(CO)_3_-Ec1 or [^125^I]I-PIB-Ec1 (1 nM) were added to cells, which were incubated at 37 °C in a humidified incubator for 1, 2, 4, 6, and 24 h. At these time points, the media were collected from one group and cells were washed once with serum-free media. To collect the membrane-bound fraction, the cells were treated with 0.2 M glycine buffer containing 4 M urea (pH 2.0) on ice for 5 min causing dissociation of membrane-bound protein. The buffer was collected, and the cells were washed once with the same buffer. Then the cells were treated with 1 M NaOH for 30 min to lyse the cells containing internalized fraction, and the solution was collected. The activity in every fraction was measured. The maximum value of cell-associated activity for each dataset was taken as 100% and the other dataset values were normalized to it. To study cellular retention and processing after interrupted incubation, the cells were incubated with [^99m^Tc]Tc(CO)_3_-Ec1 or [^125^I]I-PIB-Ec1 (10 nM) for 1 h at 4 °C. Then the media were removed, the cells were washed, fresh medium was added, and the cells were placed in a humidified incubator at 37 °C. At 1, 4, and 24 h, the medium was collected and cells were washed and treated as described above to evaluate the membrane-bound and internalized fractions.

For analysis of radiocatabolites in the supernatant after interrupted incubation, a part of it (500 µL) was separated using a NAP-5 size-exclusion column, pre-equilibrated with 1% BSA in PBS. Fractions containing activity associated with the high-molecular-weight compounds (first 900 µL) and low-molecular-weight compounds (3.6 mL) were collected. The activity in each fraction and the column were measured using a gamma-spectrometer. The residual activity left on the columns after separation was below 4% from the total activity. As a control for stability of the label, [^99m^Tc]Tc(CO)_3_-Ec1 and [^125^I]I-PIB-Ec1 were incubated in complete media at 37 °C in a humidified incubator for 24 h and analyzed as described above.

### 2.4. Affinity Measurements Using LigandTracer

The kinetics of [^125^I]I-PIB-Ec1 and [^99m^Tc]Tc(CO)_3_-Ec1 binding to living MDA-MB-468 and Ramos cells were measured using LigandTracer and evaluated using the TraceDrawer Software (both from Ridgeview Instruments, Vänge, Sweden) as described earlier [34]. Briefly, 2 × 10^6^ MDA-MB-468 cells were seeded to a local area of an 89-mm Petri dish one day before the experiment. Ramos cells growing in suspension were attached to a Petri dish following a method developed by Bondza et al. [35]. Biomolecular anchor molecule (BAM) (SUNBRIGHT^®^ OE-040CS, NOF Corporation) was dissolved in water to a concentration of 2 mg/mL. An area of a 98-mm Petri dish (Nunc, Cat No 263991) about 1.5 cm in diameter and 5 mm from the rim of the dish was covered with 400 µL of BAM solution (0.8 mg) and was incubated under sterile conditions at room temperature for 1 h. The BAM solution was aspirated and 400 µL of Ramos cell suspension (5 × 10^6^ cells/mL, 2 × 10^6^) were added dropwise to the BAM-coated area. Cells were allowed to attach to the dish for 40 min. Then the dish was tilted to remove the remaining cell suspension. The dish was covered with complete cell culture medium (10 mL) and placed into the incubator overnight. Cell attachment was confirmed the next day by observing the cells under a microscope.

To measure the binding during association phase, three concentrations of [^125^I]I-PIB-Ec1 (1.8, 5.4, and 14.5 nM) or [^99m^Tc]Tc(CO)_3_-Ec1 (0.2, 0.6, and 1.8 nM) were added to cells, followed by exchange of media and measurement of retention in the dissociation phase. Binding kinetics were recorded at room temperature and dissociation constants were calculated based on association and dissociation rates.

### 2.5. Ethical Statement

The described procedures were reviewed and approved by the Animal Research Committee at Uppsala University (ethical permission 4/16 from 26 February 2016) and were performed in accordance with the Swedish national legislation on protection of laboratory animals.

### 2.6. Animal Studies

To select an optimal label, a dual-label biodistribution study was performed. To establish MDA-MB-468 xenografts, 10^7^ cells were implanted subcutaneously in 8-week-old Balb/c nu/nu mice. For specificity control, 10^7^ EpCAM-negative Ramos cells were implanted. At the time of experimentation (two to three weeks after implantation), the weights of the animals were 17 ± 2 g in the MDA-MB-468 group and 17 ± 0 g in the Ramos group. Average tumor weights were 0.05 ± 0.04 g for MDA-MB-468 and 0.05 ± 0.04 g for Ramos. Groups of four animals per data point were used.

A well-established, dual-label approach [36,37] was selected for animal studies. In this methodology, a mixture of compounds labeled with different nuclides is co-injected into animals, and the distribution of each labeled compound is determined by gamma-spectrometry of tissue samples. A precondition for this approach is that the gamma-spectra of nuclides can be resolved. This is the case for ^125^I and ^99m^Tc (Figure S7). An advantage of the dual-label methodology is that the factors related to a host animal (e.g., individual features of metabolic rate and blood circulation) and xenografts (e.g., vascularization or presence of necrotic areas) act in the same way on both tracers. This method enables the use of a paired t-test, which provides high statistic power with a small number of animals.

Mice were injected with a mixture of both [^125^I]I-PIB-Ec1 (non-residualizing label) and [^99m^Tc]Tc(CO)_3_-Ec1 (residualizing label) and the biodistribution was measured 6 h and 24 h post injection (pi). The injected activity was 40 kBq/mouse for technetium-99m and 20 kBq/mouse for iodine-125. The injected protein dose was adjusted to 4 μg/mouse using unlabeled protein. The labeled proteins were injected into the tail vein. Before dissection, mice were anesthetized by an intraperitoneal (i.p.) injection of ketamine and xylazine solution and sacrificed by heart puncture. The dose of ketamine was 250 mg/kg and the dose of xylazine was 25 mg/kg. The organs and tissues were collected and weighed and the activity was measured using an automated gamma-spectrometer. Whole submandibular salivary gland, lung, liver, spleen, pancreas, stomach, and kidneys were sampled for measurements. A small section of small intestines was emptied of content to measure the uptake in intestinal walls. Activity in the rest of the intestinal tract was measured to estimate hepatobiliary excretion. The rest of the body was also collected and its activity was measured. The energy ranges for measurements of ^125^I and ^99m^Tc were 18–85 keV and 110–160 keV, respectively. Correction for counts’ spillover was performed automatically by the software of the gamma-spectrometer. Activity in a sample was considered as nonmeasurable (NM) if a count rate for a sample plus background was less than two-fold higher than for background (approximately 0.005% of the injected activity). The percentage of injected dose per gram of sample (%ID/g) was calculated.

In addition, an in vivo saturation experiment was performed. EpCAM receptors in MDA-MB-468 xenografts were saturated by co-injection of unlabeled protein at 0.5 mg/mouse (125-fold molar excess to 4 µg Ec1 dose used for biodistribution) together with the injection of [^125^I]I-PIB-Ec1 and [^99m^Tc]Tc(CO)_3_-Ec1, and the biodistribution measurement was performed 6 h pi. To confirm specificity, the uptake of [^125^I]I-PIB-Ec1 and [^99m^Tc]Tc(CO)_3_-Ec1 was measured in EpCAM-negative Ramos lymphoma xenografts 6 h pi.

Whole body SPECT/CT scans of mice bearing MDA-MB-468 xenografts were performed using nanoScan SPECT/CT (Mediso Medical Imaging Systems, Budapest, Hungary). Mice were injected with [^125^I]I-PIB-Ec1 (4 μg, 0.3 MBq) and [^99m^Tc]Tc(CO)_3_-Ec1 (4 μg, 9.4 MBq). Imaging at 6 h pi was performed after mice were sacrificed by CO_2_. The acquisition time was 20 min. CT scans were acquired using X-ray energy peak of 50 keV, 670 µA, 480 projections, and 5.26-min acquisition time. SPECT raw data were reconstructed using Tera-Tomo™ 3D SPECT reconstruction technology (version 3.00.020.000; Mediso Medical Imaging Systems Ltd.): High dynamic range, 30 iterations, one subset. CT data were reconstructed using Filter Back Projection and fused with SPECT files using Nucline 2.03 Software (Mediso Medical Imaging Systems Ltd.). Images are presented as maximum-intensity projections in the red, green, and blue (RGB) color scale.

To analyze the radioactive species in urine after injection of [^125^I]I-PIB-Ec1, it was injected intravenously (i.v.) into two healthy Naval Medical Research Institute (NMRI) mice (Scanbur AS, Karlslunde, Denmark) (10 µg, 1.9 MBq per mouse). Mice were kept in separate cages covered with absorbent paper, which was later checked for the activity indicating any release of urine. One hour after the injection, mice were anesthetized by i.p. injection of ketamine and xylazine solution and sacrificed by cervical dislocation. Urinary bladders containing urine were excised, cut through, the urine was collected in Eppendorf tubes, and the activity was measured (0.58 MBq in 50 µL for mouse 1; 0.68 MBq in 100 µL for mouse 2). An equal volume of ice-cold acetonitrile was added to each tube and the tubes were centrifuged at 15,000 rpm at 4 °C for 15 min. The solution was filtered through a 0.45-µm filter and analyzed by radio-HPLC. The same procedure with addition of acetonitrile and centrifugation was performed for the intact [^125^I]I-PIB-Ec1 as a control to check that it would be detectable during HPLC analysis.

Radio-HPLC analysis was performed using a Hitachi Chromaster HPLC system with a radioactivity detector and Phenomenex Luna^®^ C18 column (100 Å; 150 × 4.6 mm; 5 μm) at room temperature (20 °C). Solvent A was 0.1% trifluoroacetic acid (TFA) in H2O, solvent B was 0.1% TFA in acetonitrile, and the flow rate was 1 mL/min. The 30-min method with a gradient from 5 to 95% solvent B over 28 min and from 95% to 5% solvent B from 28 to 30 min was used.

### 2.7. Statistical Analysis

The in vitro specificity and cellular processing data are presented as the mean ± standard deviation (SD) of three samples. Statistical analysis was performed using GraphPad Prism (version 7.02; GraphPad Software, Inc., La Jolla, CA, USA). The *p* < 0.05 was considered a statistically significant difference. The data were analyzed using an unpaired two-tailed *t*-test. The biodistribution data for dual-label experiments at 6- or 24-h time points were analyzed using a paired two-tailed *t*-test.

## 3. Results

### 3.1. Radiolabeling

DARPin Ec1 was labeled site-specifically with ^99m^Tc(CO)_3_ using a hexahistidine tag at C-terminus to provide a residualizing label. Labeling of DARPin Ec1 with [^125^I]I-*para*-iodobenzoate was performed by attaching the N-hydroxysuccinimide ester derivative of [^125^I]I-PIB to amino groups of lysines. Data concerning radiolabeling of DARPin Ec1 with [^125^I]I-PIB and technetium-99m tricarbonyl are presented in Table 1. Size-exclusion chromatography provided radiochemical purities over 99%. Both labeling methods provided stable labels (Table 2 and Table 3).

### 3.2. Characterization of Radiolabeled DARPins In Vitro

**Figure 1:**
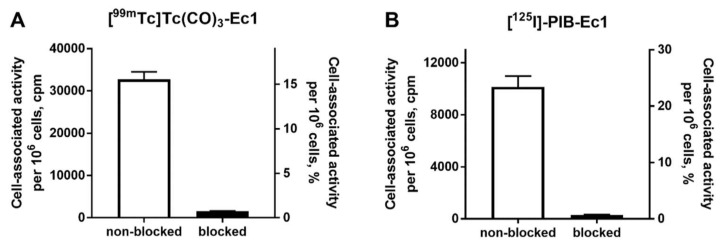
In vitro specificity of epithelial cell adhesion molecule (EpCAM) targeting using [^99m^Tc]Tc(CO)_3_-Ec1 (**A**) and [^125^I]I-PIB-Ec1 (**B**) in EpCAM-expressing MDA-MB-468 cells. Uptake by cells was significantly (*p* < 0.001) reduced when 100-fold molar excess of nonlabeled Ec1 designed ankyrin repeat protein (DARPin) was added to the blocked groups. Final concentration of radiolabeled compound was 2 nM. Data are presented as mean from three samples ± SD.

In vitro evaluation was performed using EpCAM-expressing MDA-MB-468 breast cancer cells. To demonstrate binding specificity of [^125^I]I-PIB-Ec1 and [^99m^Tc]Tc(CO)_3_-Ec1 to EpCAM, the EpCAM receptors were saturated with 100-fold molar excess of nonlabeled Ec1 before addition of the radiolabeled compound. Blocking the EpCAM receptors resulted in a significant (*p* < 0.001) decrease of both [^125^I]I-PIB-Ec1 and [^99m^Tc]Tc(CO)_3_-Ec1 uptake (Figure 1). This demonstrated a saturable character of radiolabeled Ec1 binding to MDA-MB-468 cells.

**Figure 2:**
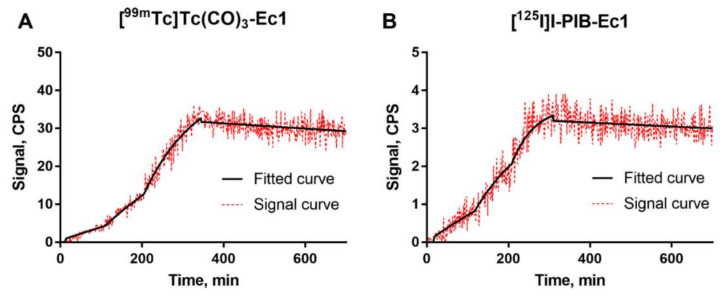
LigandTracer sensorgrams of [^99m^Tc]Tc(CO)_3_-Ec1 (**A**) and [^125^I]I-PIB-Ec1 (**B**) binding to MDA-MB-468 cells. The association was measured at 0.2, 0.6, and 1.8 nM concentrations for [^99m^Tc]Tc(CO)_3_-Ec1 and at 1.8, 5.4, and 14.5 nM concentrations [^125^I]I-PIB-Ec1.

The binding kinetics of [^99m^Tc]Tc(CO)_3_-Ec1 and [^125^I]I-PIB-Ec1 to MDA-MB-468 cells were measured using LigandTracer (Figure 2, Figures S1 and S2). A rapid binding and slow dissociation were observed. The equilibrium dissociation constant (K_D_) values for both probes were in the picomolar range (Table 1). As a control for nonspecific interactions with cells, binding of [^99m^Tc]Tc(CO)_3_-Ec1 and [^125^I]I-PIB-Ec1 to EpCAM-negative Ramos cells was also measured using LigandTracer (Figures S3 and S4). The signal detected from Ramos cells was comparable to the background and it was much lower than the signal detected from MDA-MB-468 cells.

**Figure 3:**
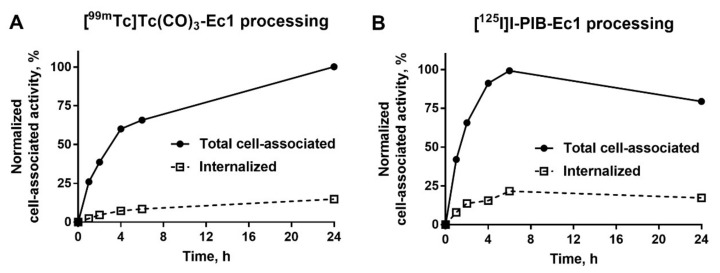
Cellular processing of [^99m^Tc]Tc(CO)_3_-Ec1 (**A**) and [^125^I]I-PIB-Ec1 (**B**) by MDA-MB-468 cells during continuous incubation. Cells were incubated with the DARPins (1 nM) at 37 °C. Data are presented as the mean of three samples ± standard deviation (SD). Error bars might not be seen when they are smaller than data point symbols.

The processing of [^99m^Tc]Tc(CO)_3_-Ec1 and [^125^I]I-PIB-Ec1 by MDA-MB-468 breast cancer cells during continuous incubation is shown in Figure 3. For [^99m^Tc]Tc(CO)_3_-Ec1, the total cell-associated activity increased continuously over 24 h incubation and the internalized fraction had also a tendency to a slow increase. For [^125^I]I-PIB-Ec1, the maximum of total cell-associated activity was reached at 6 h and it slowly decreased by 24 h. This could be explained by the non-residualizing properties of the [^125^I]I-PIB label and the diffusion of iodine catabolites from the cells after internalization. A characteristic feature of radiolabeled Ec1 was the quite low internalization. The internalized fraction for [^99m^Tc]Tc(CO)_3_-Ec1 was approximately 15% of the total cell-associated activity at 24 h.

**Figure 4:**
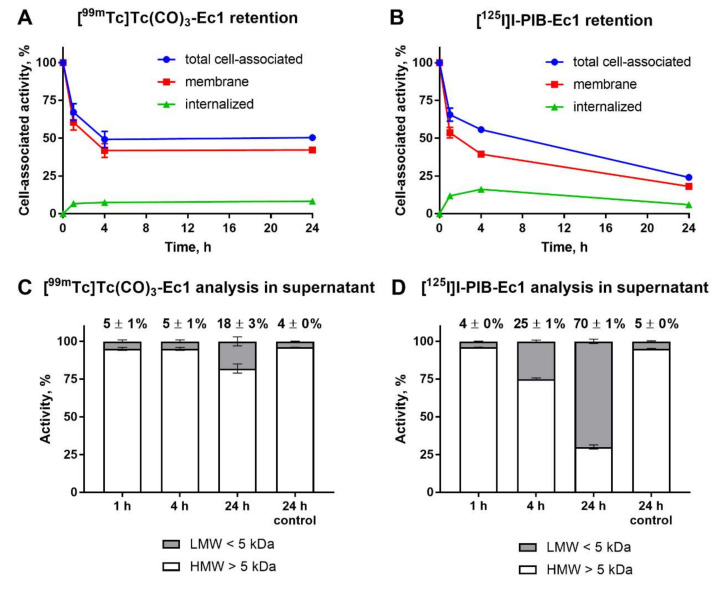
Cellular retention of activity after interrupted incubation of [^99m^Tc]Tc(CO)_3_-Ec1 (**A**) and [^125^I]I-PIB-Ec1 (**B**) with MDA-MB-468 cells. Cells were first incubated with the DARPin variants (10 nM) at 4 °C for 1 h and then the media were exchanged and the cells were incubated at 37 °C for 1, 4, or 24 h. A fraction of the supernatant at every time point was analyzed using NAP-5 size-exclusion columns and compared to the control when the radiolabeled DARPins were incubated in complete media for 24 h (**C**,**D**). Numbers in panels (**C**,**D**) show a percentage of activity associated with low-molecular-weight compounds at each time point. Data for retention are presented as the mean of three samples ± SD and data for NAP-5 analysis are presented as the mean of two samples ± SD. LMW = low molecular weight, HMW = high molecular weight. Error bars might not be seen when they are smaller than data point symbols.

Cellular processing and retention of [^99m^Tc]Tc(CO)_3_-Ec1 and [^125^I]I-PIB-Ec1 by MDA-MB-468 cells after interrupted incubation is shown in Figure 4. After initial release of both probes from the membranes due to the shift in equilibrium, the cell-associated activity for [^99m^Tc]Tc(CO)_3_-Ec1 remained constant until 24 h (Figure 4A). On the opposite, the cell-associated activity continued to decrease in the case of [^125^I]I-PIB-Ec1 (Figure 4B). This decrease was associated with the gradual build-up of low-molecular-weight (<5 KDa) radioactive compounds in the media (25 ± 1% at 4 h and 70 ± 1% at 24 h) (Figure 4D). For [^99m^Tc]Tc(CO)_3_-Ec1, the low-molecular-weight activity fraction was only 18 ± 3% at 24 h. Most likely, these low-molecular-weight compounds are the products of intracellular degradation released from cells, since only less than 5% of activity was in the low-molecular-weight fraction after 24-h incubation of both compounds in cell-free complete media. The difference in the amount of low-molecular-weight-associated activity reflects, most likely, the difference in intracellular retention of radiometabolites of iodine and technetium labels. Identity and purity of the radiolabeled [^99m^Tc]Tc(CO)_3_-Ec1 and [^125^I]I-PIB-Ec1 was confirmed by radio-HPLC analysis (Figure S5).

### 3.3. In Vivo Studies

**Figure 5:**
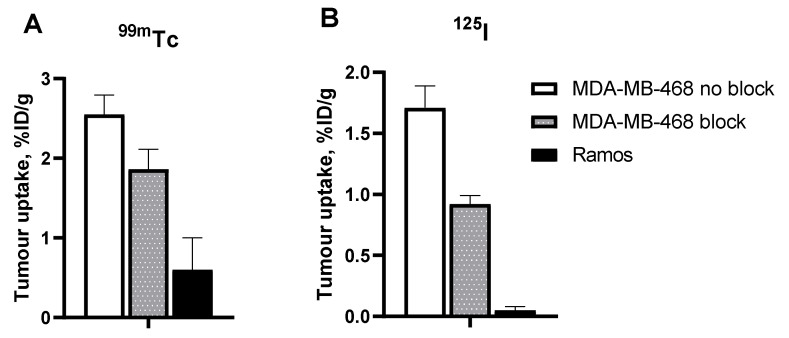
In vivo specificity of EpCAM targeting using [^99m^Tc]Tc(CO)_3_-Ec1 (**A**) and [^125^I]I-PIB-Ec1 (**B**). Uptake of both DARPin variants was significantly (*p* < 0.01, unpaired *t*-test) higher in EpCAM-positive MDA-MB-468 xenografts than in EpCAM-negative Ramos xenografts 6 h post injection (pi). EpCAM blocking in MDA-MB-468 xenografts by co-injecting a large excess of unlabeled Ec1 also resulted in a significant decrease of tracer uptake. Data are presented as mean ± SD for four mice.

The results of the specificity test (Figure 5) demonstrated that the uptake of both [^99m^Tc]Tc(CO)_3_-Ec1 and [^125^I]I-PIB-Ec1 in EpCAM-negative Ramos xenografts was much lower (*p* < 0.001, unpaired t-test) than in EpCAM-positive MDA-MB-468 xenografts. In addition, saturation of EpCAM by co-injecting a large excess of unlabeled Ec1 resulted in a significant (*p* < 0.01, unpaired *t*-test) reduction in tumor uptake of both variants.

The tumor uptake and retention of [^99m^Tc]Tc(CO)_3_-Ec1 was significantly (*p* < 0.05, paired *t*-test) higher than tumor uptake and retention of [^125^I]I-PIB-Ec1 (2.6 ± 0.2 vs. 1.7 ± 0.2%ID/g 6 h pi). Distribution of activity in normal organs and tissues was quite different for ^99m^Tc- and ^125^I-labeled Ec1 (Table 4). The renal uptake of [^99m^Tc]Tc(CO)_3_-Ec1 was more than 100-fold higher compared to [^125^I]I-PIB-Ec1 even 24 h pi. A combination of a high kidney retention of activity and low activity accumulation in gastrointestinal tract indicated a renal excretion pathway of DARPin Ec1. The renal and hepatic uptake of [^99m^Tc]Tc(CO)_3_-Ec1 was higher than the tumor uptake. [^125^I]I-PIB-Ec1 had appreciably lower hepatic and renal uptake. Overall, [^125^I]I-PIB-Ec1 had the lowest uptake in normal tissues. Already by 6 h after injection of [^125^I]I-PIB-Ec1 only ca. 4% of ID was left in mice and over 95% of ID was excreted in comparison with [^99m^Tc]Tc(CO)_3_-Ec1, when about 30% of ID was excreted by 6 h (Table S1). Accordingly, [^125^I]I-PIB-Ec1 provided significantly higher tumor-to-organ ratios compared with [^99m^Tc]Tc(CO)_3_-Ec1 (Table 5). At 6 h pi, [^125^I]I-PIB-Ec1 had two-fold higher tumor-to-blood, ca. 100-fold higher tumor-to-liver, 20-fold higher tumor-to-spleen and tumor-to-pancreas, and eight-fold higher tumor-to-muscle ratios than [^99m^Tc]Tc(CO)_3_-Ec1. By 24 h pi, the tumor-to-blood and tumor-to-kidney ratios increased further for the [^125^I]I-PIB label, while no improvement was observed for the technetium-99m label.

To study if the lower activity retention in kidneys in case of [^125^I]I-PIB-Ec1 is caused by the excretion of radiometabolites, we performed radio-HPLC analysis of urine collected 1 h after [^125^I]I-PIB-Ec1 injection in healthy NMRI mice (Figure S6). It was observed that the majority of activity excreted with urine was in the form of radiocatabolites already 1 h after the injection of [^125^I]I-PIB-Ec1.

**Figure 6:**
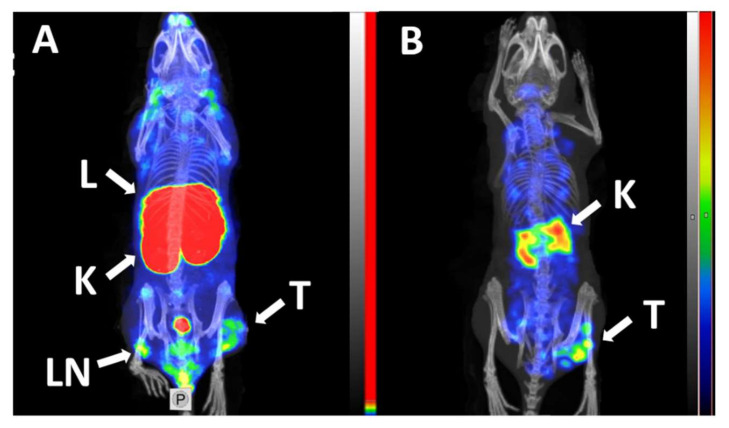
Micro-Single-Photon Emission Computed Tomography/Computed Tomography (microSPECT/CT) imaging of EpCAM expression in BALB/C nu/nu mice bearing EpCAM-positive MDA-MB-468 xenografts at 6 h pi using [^99m^Tc]Tc(CO)_3_-Ec1 (**A**) and [^125^I]I-PIB-Ec (**B**). Arrows indicate: T—tumor, K—kidneys, L—liver, LN—lymph node. The scale in panel A was adjusted to the first red pixel in the tumor.

MicroSPECT/CT imaging using [^99m^Tc]Tc(CO)_3_-Ec1 and [^125^I]I-PIB-Ec in Balb/c nu/nu mice bearing MDA-MB-468 xenografts at 6 h pi confirmed the results of the biodistribution studies (Figure 6). The tumor was visualized using both radiolabeled variants of DARPin Ec1. The use of non-residualizing label [^125^I]I-PIB-Ec1 provided lower retention of activity in normal organs and kidneys and higher imaging contrast compared to [^99m^Tc]Tc(CO)_3_-Ec1.

## 4. Discussion

Efficacy of targeted therapies is critically dependent on expression level of the molecular target in tumors. In the case of absence or too low expression level of the target, there will be no therapeutic effect. Unfortunately, an unspecific toxicity to normal organs and tissues will be preserved. This is a particularly apparent risk for a targeted delivery of cytotoxic payloads, such as drugs, toxins, or alpha or beta particle-emitting radionuclides. Thus, personalizing treatment by stratification of patients according to expression of a molecular target in tumors is an essential precondition for successful targeted therapy. Although the percentage of EpCAM-overexpressing TNBC tumors is high, identification of patients eligible for therapy is necessary to avoid overtreatment of patients having tumors with low expression level. Radionuclide molecular imaging is a promising approach, as it enables visualization of multiple metastases addressing heterogeneity of expression.

Imaging data can be used to select patients for targeted therapy in different ways. The most common method is the determination of tumor-to-reference organ ratio. A typical example is the so-called Krenning score, which is applied for selection of patients with neuroendocrine tumors for somatostatin receptor-targeted radionuclide therapy using [^177^Lu-DOTA^0^,Tyr^3^]octreotate [38,39]. The scale grades the tumor uptake of [^111^ In-DTPA^0^]octreotide in planar gamma camera images: Grade 1, no tumor uptake; grade 2, tumor uptake is equal to normal liver tissue; grade 3, tumor uptake is greater than normal liver tissue; and grade 4, tumor uptake is higher than normal spleen or kidney uptake. It has been demonstrated that the response strongly correlates with the grade [38]. It has been shown that tumor-to-spleen uptake ratio for ^111^In and ^68^Ga-labeled affibody molecules [40] and tumor-to-contralateral breast ratio for ^99m^Tc-labeled ADAPT6 [41] correlates strongly with the level of HER2 expression in breast cancer. Alternatively, the uptake of an imaging probe in tumor might be quantified using PET and correlated with expression level [42].

An essential factor for successful radionuclide imaging is high imaging contrast because it determines the diagnostic sensitivity. Thus, imaging agents providing high tumor-to-organ ratios (ratios of activity concentration in tumors to the concentration in normal organs), especially to organs that are frequent metastatic sites, are required. TNBC has a predominant metastasis to visceral organs, first and foremost, liver and lungs [5,43]. Thus, sufficiently high tumor-to-liver and tumor-to-lung ratios are the preconditions for successful translation to clinics. In addition, a high tumor-to-blood ratio is an essential parameter for evaluation of an imaging agent as a blood-borne activity and might contribute to the background signal.

Selection of a fitting label is important for obtaining of a high contrast. One strategy to achieve a high imaging contrast is based on using non-residualizing labels. After binding of radiolabeled proteins to cell-surface receptors on cancer cells or to scavenger receptors on cells in excretory organs (e.g., kidneys and liver), a protein-receptor complex is internalized and the radiolabeled protein is degraded in lysosomes with formation of radiocatabolites. Radiocatabolites of residualizing labels (typically, radiometals) are retained inside the cells as they are not able to diffuse through the lipophilic membranes. However, the radiocatabolites of non-residualizing labels rapidly diffuse from the cells, return to blood circulation, and are excreted with urine. When the internalization of radiolabeled proteins is slow in tumors and rapid in normal organs and tissues, the use of non-residualizing labels might enable fast clearance of activity from normal organs and tissues and provide higher imaging contrast than the use of residualizing labels.

Three different variants of non-residualizing radioiodine labels were evaluated for DARPins: A product of so-called direct electrophilic radioiodination, when the radioiodine is incorporated into the phenolic ring of tyrosine [22,23,30], a conjugate with [^125^I]-PIB [30,31], and a site-specific conjugate of [^125^I]I-iodo-[(4-hydroxyphenyl)ethyl]-maleimide (HPEM) to a unique cysteine engineered to a C-terminus of DARPin G3 [24]. It turned out that the use of the HPEM label results in a high level of hepatobiliary excretion and accumulation of activity in the content of gastrointestinal tract. This is undesirable, as it creates an unacceptable high background for imaging of visceral TNBC metastases. The use of direct iodination resulted in accumulation of radiocatabolites in Na/I-symporter-expressing organs (first and foremost, in salivary gland and stomach) but also in in visceral organs such as pancreas and intestines [30]. Overall, previous studies suggest that indirect radioiodination using PIB would be optimal for labeling of Ec1.

A potential disadvantage of the use of [^125^I]I-PIB is a random attachment of N-succinimidyl-*para*-iodobenzoate to amino groups of lysines in DARPin Ec1. As DARPin Ec1 contains eight lysines, this labeling might result in a mixture of labeled proteins with a different number and positions of [^125^I]I-PIB label. However, previous studies with proteins of similar size showed that the specificity and affinity is usually preserved with this type of labeling [27,44,45,46].

This study showed that the binding of both [^125^I]I-PIB-Ec1 and [^99m^Tc]Tc(CO)_3_-Ec1 to TNBC cells in vitro (Figure 1) can be blocked with an excess of unlabeled Ec1, which confirmed its specificity.

Previous studies have demonstrated that kinetics and affinity of binding of radiolabeled proteins to their molecular targets depend on cell line origin [47,48]. This might be associated with the molecular context of cellular membranes, including such factors as co-expression of other receptors or cell-surface proteins, glycosylation patterns, and homo- and heterodimerization. The results of LigandTracer measurements (Figure 2) demonstrated that binding of both tracers to TNBC cells was characterized by rapid association and very slow dissociation rates. The equilibrium dissociation constants were 121 ± 21 pM and 58 ± 5 pM for [^125^I]I-PIB-Ec1 and [^99m^Tc]Tc(CO)_3_-Ec1, respectively. Thus, affinity of [^125^I]I-PIB-Ec1 binding to TNBC cells was somewhat lower compared to binding to ovarian cancer OvCAR-3 cells (35 ± 1 pM) [31] or pancreatic cancer BxPC-3 cells (58 ± 13 pM) [30]. According to stoichiometric calculations, the average number of pendant groups conjugated per Ec1 molecule should be 0.3, which should have a minimal impact on binding properties. However, it might happen that one of the pendant groups is conjugated to a lysine close to the binding site and might create a hindrance. This might explain the differences in affinities between [^99m^Tc]Tc(CO)_3_-Ec1, which was labeled site specifically, and [^125^I]I-PIB-Ec1. Still, the picomolar affinity is a good precondition for a strong retention of an imaging probe by malignant cells in vivo.

Assessment of the internalization rate of Ec1 during continuous incubation of cells with the tracer was initially performed using [^99m^Tc]Tc(CO)_3_-Ec1 (Figure 3). A residualizing label is the most suitable for such kind of test because the data obtained by a non-residualizing label might be deceptive due to the “leakage” of radiometabolites from cells leading to underestimation of internalized radioactivity. The cellular processing study showed a slow internalization (approximately 15% of cell-bound activity at 24 h after incubation start) of Ec1 by TNBC cells. The difference between cellular processing of [^99m^Tc]Tc(CO)_3_-Ec1 and [^125^I]I-PIB-Ec1 was further elucidated by studying cellular retention after interrupted incubation (Figure 4). The use of radioiodine label was associated with lower retention of activity by cells. In addition, a decrease of cell-associated radioiodine activity was accompanied with an increase of low-molecular-weight radioiodinated compounds in the incubation media. Most likely, this was caused by diffusion of radiometabolites of a non-residualizing label from cells. We considered the combination of the slow internalization and high affinity as a good rationale to proceed with in vivo studies.

Animal studies demonstrated clearly specific accumulation of both [^125^I]I-PIB-Ec1 and [^99m^Tc]Tc(CO)_3_-Ec1 in MDA-MB-468 xenografts (Figure 5). Saturation of EpCAM by co-injection of a large excess of unlabeled Ec1 resulted in significantly lower uptake of the tracers in TNBC xenografts. In addition, the activity uptake in EpCAM-negative Ramos xenografts was much lower than in EpCAM-positive MDA-MB-468 xenografts.

Low accumulation in normal tissue is an important precondition for a high-contrast imaging. The results of the biodistribution experiments demonstrated advantages in the use of non-residualizing labels for slowly internalizing, high-affinity imaging probes (Table 4). The difference in the renal uptake of [^125^I]I-PIB-Ec1 and [^99m^Tc]Tc(CO)_3_-Ec1 was spectacular already at 6 h after injection. A high re-absorption in the proximal tubuli of the kidneys is a common feature of imaging probes based on short peptides [49] and ESPs, such as affibody molecules, ADAPTs, or DARPins [20,50,51]. In the case of short peptides, the re-absorption could be suppressed by blocking scavenger receptors with cationic amino acids or succinylated bovine gelatin (Gelofusine) [49]. However, the use of these and a number of other substances potentially blocking or reducing renal reabsorption was unsuccessful in the case of DARPins [51]. Thus, there is no conventional way to reduce the renal uptake of DARPins labeled with a residualizing label. However, the use of [^125^I]I-PIB-Ec1 resulted in more than a 70-fold lower renal uptake compared with [^99m^Tc]Tc(CO)_3_-Ec1. Radio-HPLC analysis of the urine 1 h after injection of [^125^I]I-PIB-Ec1 demonstrated that the activity was excreted predominantly as radiometabolites (S6). This suggests that non-residualizing properties of the radioiodine label were critical for the reduction of renal retention. Even more impressive (and more relevant to imaging of EpCAM expression in TNBC metastases) was the reduction of hepatic uptake, which was 160-fold lower for [^125^I]I-PIB-Ec1. While the uptake of [^99m^Tc]Tc(CO)_3_-Ec1 in tumor was nearly 7-fold lower than in liver, the tumor uptake of [^125^I]I-PIB-Ec1was 15-fold higher than the hepatic uptake.

It has to be noted that the tumor uptake of [^99m^Tc]Tc(CO)_3_-Ec1 was significantly (*p* < 0.05, paired *t*-test) higher than tumor uptake and retention of [^125^I]I-PIB-Ec1 (2.6 ± 0.2 vs. 1.7 ± 0.2%ID/g 6 h pi). This can be explained by the lower affinity of [^125^I]I-PIB-Ec1 compared with its ^99m^Tc-labeled counterpart. In addition, we can expect that some internalization would take place during six hours and leakage of catabolites would affect the retention of activity in tumors. However, the magnitude of the uptake reduction was appreciably smaller compared with the uptake in normal tissues. For this reason, tumor-to-organ ratios were several folds higher for [^125^I]I-PIB-Ec1 than for [^99m^Tc]Tc(CO)_3_-Ec1 (Table 3). Particularly, tumor-to-blood, tumor-to-lung, tumor-to-liver, and tumor-to-muscle ratios were 19 ± 3, 8 ± 2, 15 ± 2, and 42 ± 10 at 6 h after injection, respectively. Accordingly, an experimental microSPECT imaging using [^125^I]I-PIB-Ec1 permitted clear visualization of EpCAM expression in TNBC xenografts (Figure 5). It has to be noted that the tumor-associated activity decreased with time after injection of [^125^I]I-PIB-Ec1. At 24 h pi, the tumor uptake of [^125^I]I-PIB-Ec1was only 0.27 ± 0.05%ID/g. This was more than five-fold lower compared with the tumor uptake of [^99m^Tc]Tc(CO)_3_-Ec1. Such low uptake makes this tracer unsuitable for imaging the next day after injection. Thus, non-residualizing labels are suitable for imaging only a few hours after injection even in the cases of slow internalization by malignant cells.

It should also be noted that the observed differences in the biodistribution between [^99m^Tc]Tc(CO)_3_-Ec1 and [^125^I]I-PIB-Ec1 might not only be due to residualizing properties of labels but also be influenced by other label properties (polarity, site specificity of labeling), as well as by differences in pharmacokinetics.

In this study, ^125^I was used as a label because of its convenient half-life (60 days). However, its low-energy electromagnetic radiation (max. 35.5 keV) permits its use only in small rodents and is unsuitable for clinical translation. Two iodine radioisotopes, ^123^I (T_1/2_ = 13.3 h, Eγ = 159 keV) and positron-emitting ^124^I (T_1/2_ = 100 h, β+ 23%), are suitable for radionuclide imaging using single-photon computed tomography (SPECT) and positron emission tomography (PET), respectively. Since the chemical properties of isotopes are identical, only a minor re-optimization is required to change the labeling chemistry from ^125^I to these nuclides [24,52].

Summarizing, this study demonstrated that [^125^I]I-PIB-Ec1 has high (subnanomolar) affinity to EpCAM. It binds in a specific manner to EpCAM-expressing TNBC cell line in vitro and accumulates specifically in EpCAM-expressing TNBC xenografts in mice. The tumor-to-organ ratios, which are a measure of imaging contrast and sensitivity, are appreciably higher for [^125^I]I-PIB-Ec1 than for [^99m^Tc]Tc(CO)_3_-Ec1. The [^125^I]I-PIB-Ec1 is capable of visualization of EpCAM-expressing TNBC xenograft in a mouse. This creates a rationale for further clinical studies concerning imaging of EpCAM in TNBC and correlation of imaging and biopsy data.

## 5. Conclusions

Both [^125^I]I-PIB-Ec1 and [^99m^Tc]Tc(CO)_3_-Ec1 demonstrated specific uptake in EpCAM-positive TNBC xenografts. Radioiodine provided better tumor-to-organ ratios compared to [^99m^Tc]Tc(CO)_3_ label. Radioiodinated DARPin Ec1 is a promising agent for same-day imaging of EpCAM expression in triple-negative breast cancer using SPECT.

## Figures and Tables

**Table 1:** Labeling and characterization of radiolabeled Ec1 variants.

DARPins	Radiochemical Yield of Non-Isolated Compound (%)	Radiochemical Yield of Isolated Compound (%)	Radiochemical Purity (%)	Binding Affinity to MDA-MB-468 Cells (K_D_, pM)
[^125^I]I-PIB-Ec1	23 ± 2 (*n* = 3)	19 ± 1 (*n* = 3)	99 ± 0 (*n* = 3)	121 ± 21 (*n* = 2)
[^99m^Tc]Tc(CO)_3_-Ec1	92 ± 1 (*n* = 3)	69 ± 7 (*n* = 3)	99 ± 0 (*n* = 3)	58 ± 5 (*n* = 2)

**Table 2:** In vitro stability of [^99m^Tc]Tc(CO)_3_-Ec1.

	Protein-Associated Activity, %	
	1000× Histidine	PBS
1 h	99 ± 0	99 ± 0
4 h	99 ± 0	99 ± 1
24 h	98 ± 0	99 ± 1

Samples were incubated in PBS or with 1000-fold molar excess of histidine at 37 °C. Analysis was performed in duplicates.

**Table 3:** In vitro stability of [^125^I]I-PIB-Ec1.

	Protein-Associated Activity, %		
	1000× NaI	30% EtOH	PBS
1 h	98 ± 1	99 ± 0	99 ± 0
4 h	99 ± 0	99 ± 0	99 ± 0
24 h	99 ± 0	98 ± 0	99 ± 0

Samples were incubated in PBS, 30% ethanol or with 1000-fold molar excess of NaI at 37 °C. Analysis was performed in duplicates.

**Table 4:** Biodistribution of [^99m^Tc]Tc(CO)_3_-Ec1 and [^125^I]I-PIB-Ec1 in Balb/c nu/nu mice bearing MDA-MB-468 xenografts 6 and 24 h pi.

Tissue	[^99m^Tc]Tc(CO)_3_-Ec1	[^125^I]I-PIB-Ec1	[^99m^Tc]Tc(CO)_3_-Ec1	[^125^I]I-PIB-Ec1
	6 h		24 h	
Blood	0.24 ± 0.03 *^a,b^*	0.09 ± 0.01 *^c^*	0.11 ± 0.01 *^a^*	0.009 ± 0.001
Salivary glands	1.7 ± 0.2 *^a^*	0.11 ± 0.04	1.3 ± 0.3	NM
Lungs	0.9 ± 0.2 *^a,b^*	0.23 ± 0.03 *^c^*	0.6 ± 0.2 *^a^*	0.04 ± 0.01
Liver	18 ± 2 *^a,b^*	0.11 ± 0.02 *^c^*	9 ± 2 *^a^*	0.026 ± 0.002
Spleen	3.1 ± 0.3 *^a,b^*	0.10 ± 0.02 *^c^*	2.2 ± 0.3	0.044 ± 0.003
Pancreas	1.2 ± 0.2 *^a,b^*	0.04 ± 0.01	0.8 ± 0.2	NM
Small intestine	1.5 ± 0.3 *^a,b^*	0.11 ± 0.04	0.9 ± 0.3	NM
Stomach	1.8 ± 0.4 *^a,b^*	0.15 ± 0.03	0.9 ± 0.2	NM
Kidney	192 ± 15 *^a,b^*	2.7 ± 1.0 *^c^*	114 ± 13 *^a^*	0.08 ± 0.01
Tumor	2.6 ± 0.2 *^a^*	1.7 ± 0.2 *^c^*	1.5 ± 0.5 *^a^*	0.27 ± 0.05
Muscle	0.5 ± 0.1 *^a,b^*	0.04 ± 0.01	0.3 ± 0.1	NM
Bone	1.9 ± 0.3 *^a,b^*	0.9 ± 0.2 *^c^*	1.2 ± 0.3 *^a^*	0.5 ± 0.2
Intestines with content	1.8 ± 0.3 *^b^*	0.4 ± 0.1 *^c^*	1.1 ± 0.1 *^a^*	0.07 ± 0.01
Rest of the body	11.6 ± 1.2 *^a,b^*	1.7 ± 0.2 *^c^*	8.5 ± 1.5 *^a^*	0.9 ± 0.2

Data are presented as mean percent of injected dose (%ID)/g ± SD for four mice. Data for the rest of the intestines with contents and rest of the body are presented as %ID per whole sample. *^a^* Significant difference between [^99m^Tc]Tc and [^125^I]I at the same time point (paired t-test). *^b^* Significant difference between values for [^99m^Tc]Tc at 6- and 24-h time point (unpaired t-test). *^c^* Significant difference between values for [^125^I]I at 6- and 24-h time point (unpaired *t*-test). NM = nonmeasurable.

**Table 5:** Tumor-to-organ ratios of [^99m^Tc]Tc(CO)_3_-Ec1 and [^125^I]I-PIB-Ec1 in Balb/C nu/nu mice bearing MDA-MB-468 xenografts at 6 and 24 h pi.

Tissue	[^99m^Tc]Tc(CO)_3_-Ec1	[^125^I]I-PIB-Ec1	[^99m^Tc]Tc(CO)_3_-Ec1	[^125^I]I-PIB-Ec1
	6 h		24 h	
Blood	11 ± 1 *^a^*	19 ± 3 *^c^*	13 ± 4 *^a^*	31 ± 6
Salivary glands	1.5 ± 0.1 *^a^*	17 ± 6	1.2 ± 0.3	NM
Lungs	3 ± 1 *^a^*	8 ± 2	3 ± 1	NM
Liver	0.14 ± 0.01 *^a^*	15 ± 2 *^c^*	0.2 ± 0.1 *^a^*	10 ± 1
Spleen	0.8 ± 0.1 *^a^*	18 ± 5	0.7 ± 0.2	NM
Pancreas	2.1 ± 0.2 *^a^*	43 ± 8	2 ± 1	NM
Small intestine	1.7 ± 0.4 *^a^*	17 ± 6	2 ± 1	NM
Stomach	1.4 ± 0.3 *^a^*	12 ± 4	2 ± 1	NM
Kidney	0.013 ± 0.002 *^a^*	0.7 ± 0.3 *^c^*	0.014 ± 0.005 *^a^*	3.5 ± 0.5
Muscle	5 ± 1 *^a^*	42 ± 10	5 ± 1	NM
Bone	1.3 ± 0.2	2 ± 1 *^c^*	1.3 ± 0.4	0.5 ± 0.1

Data are presented as mean ± SD for four mice. *^a^* Significant difference between [^125^I]I and [^99m^Tc]Tc at the same time point (paired t-test). *^b^* Significant difference between values for [^99m^Tc]Tc at 6- and 24-h (unpaired t-test). *^c^* Significant difference between values for [^125^I]I at 6- and 24-h (unpaired *t*-test). NM= nonmeasurable.

## Supplementary Materials

The following are available online, Figure S1: representative curves of the LigandTracer measurement of [^99m^Tc]Tc(CO)_3_-Ec1 binding to MDA-MB-468 cells, Figure S2: representative curves of the LigandTracer measurement of [^125^I]I-PIB-Ec1 binding to MDA-MB-468 cells, Figure S3: representative curves of the LigandTracer measurement of [^99m^Tc]Tc(CO)_3_-Ec1 binding to Ramos cells, Figure S4: representative curves of the LigandTracer measurement of [^125^I]I-PIB-Ec1 binding to Ramos cells; Figure S5: radio-HPLC analysis of [^125^I]I-PIB-Ec1 and [^99m^Tc]Tc(CO)_3_-Ec1 in comparison to the non-labeled Ec1, Figure S6: radio-HPLC analysis of mouse urine 1 h after injection of [^125^I]I-PIB-Ec1 in comparison to the intact [^125^I]I-PIB-Ec1, Figure S7: resolution of gamma-spectra of iodine-125 and technetium-99m, Table S1: biodistribution of [^99m^Tc]Tc(CO)_3_-Ec1 and [^125^I]I-PIB-Ec1 in Balb/c nu/nu mice bearing MDA-MB-468 xenografts at 6 and 24 h.
