# Supplementary material for: Radionuclide Molecular Imaging of EpCAM Expression in Triple-Negative Breast Cancer Using the Scaffold Protein DARPin Ec1
Source: Molecules. 2020 Oct 14;25(20):4719. doi: 10.3390/molecules25204719 (PMC7587533; doi:10.3390/molecules25204719)
Supplement: Supplementary file 1 [file molecules-25-04719-s001.pdf]

Supplementary Materials

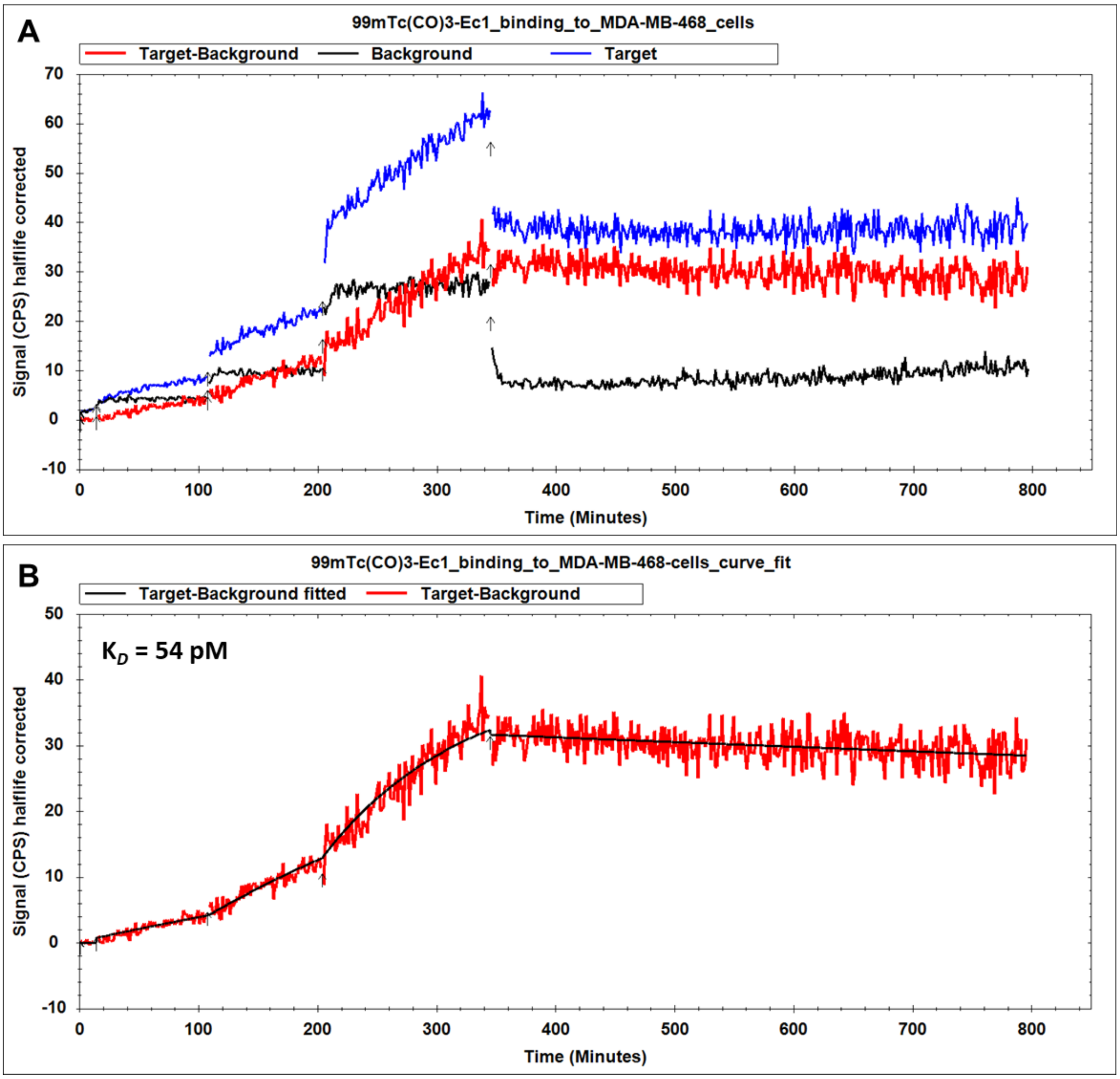

Figure S1. Representative curves of the LigandTracer measurement of [^{99m}Tc]Tc(CO)$_3$-Ec1 binding to MDA-MB-468 cells. The signal curves display every data point. (**A**) Signal from the cell-free reference area (plastic area of a Petri dish) is shown in black (Background). Signal from the area with cells is shown in blue (Target). Subtraction of the Background signal from Target signal is shown in red (Target-Background). (**B**) The signal corrected for background (Target-Background, red) was analyzed by the TraceDrawer software using Langmuir's 1:1 interaction model, the fitted curve is shown in black. Specific activity was 1.4 MBq/µg (start of experiment). The association was measured at 0.2, 0.6 and 1.8 nM concentrations.

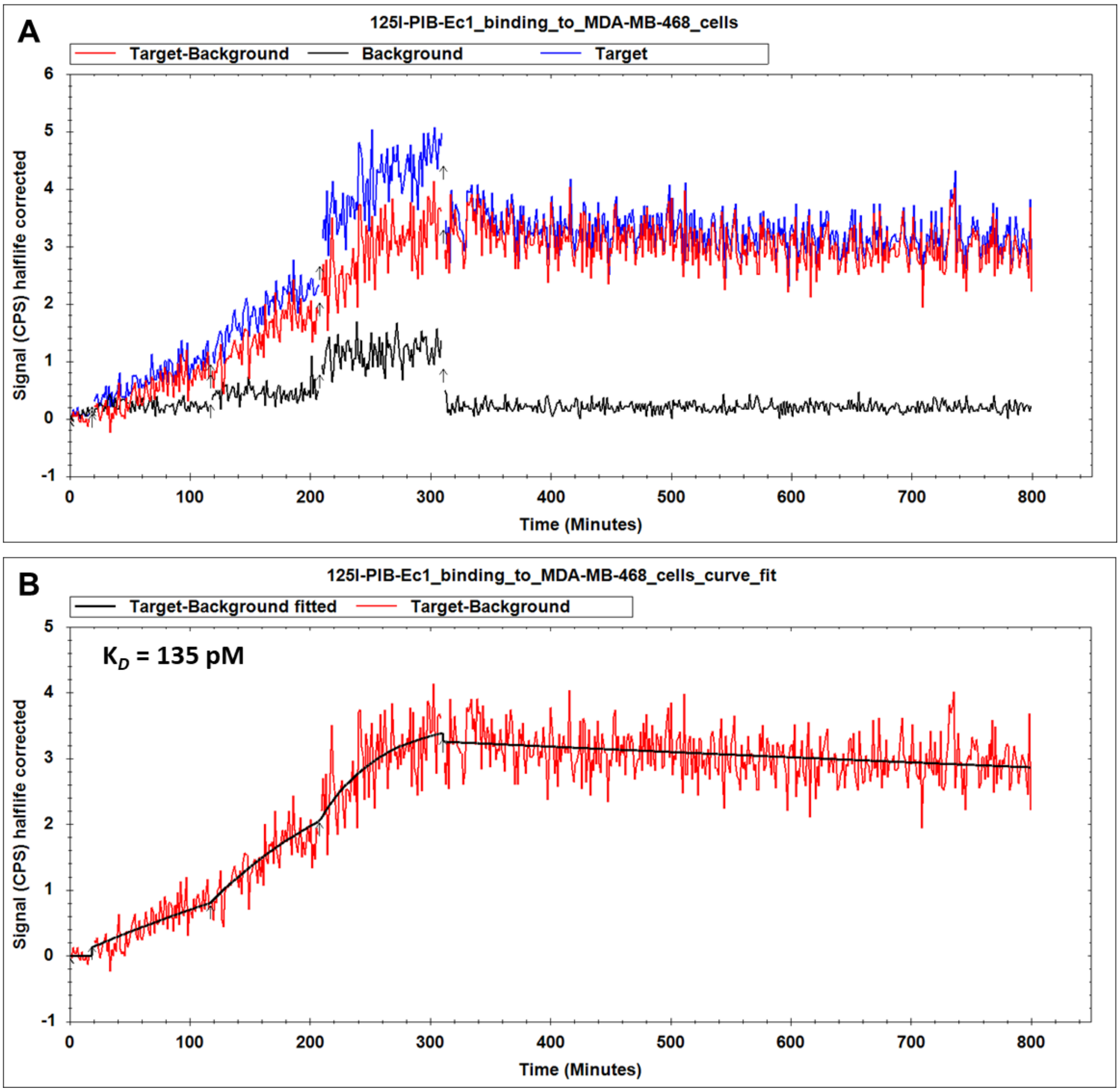

Figure S2. Representative curves of the LigandTracer measurement of [^{125}I]I-PIB-Ec1 binding to MDA-MB-468 cells. The signal curves display every data point. (**A**) Signal from the cell-free reference area (plastic area of a Petri dish) is shown in black (Background). Signal from the area with cells is shown in blue (Target). Subtraction of the Background signal from Target signal is shown in red (Target-Background). (**B**) The signal corrected for background (Target-Background, red) was analyzed by the TraceDrawer software using Langmuir's 1:1 interaction model, the fitted curve is shown in black. Specific activity was 0.03 MBq/µg. The association was measured at 1.8, 5.4 and 14.5 nM concentrations.

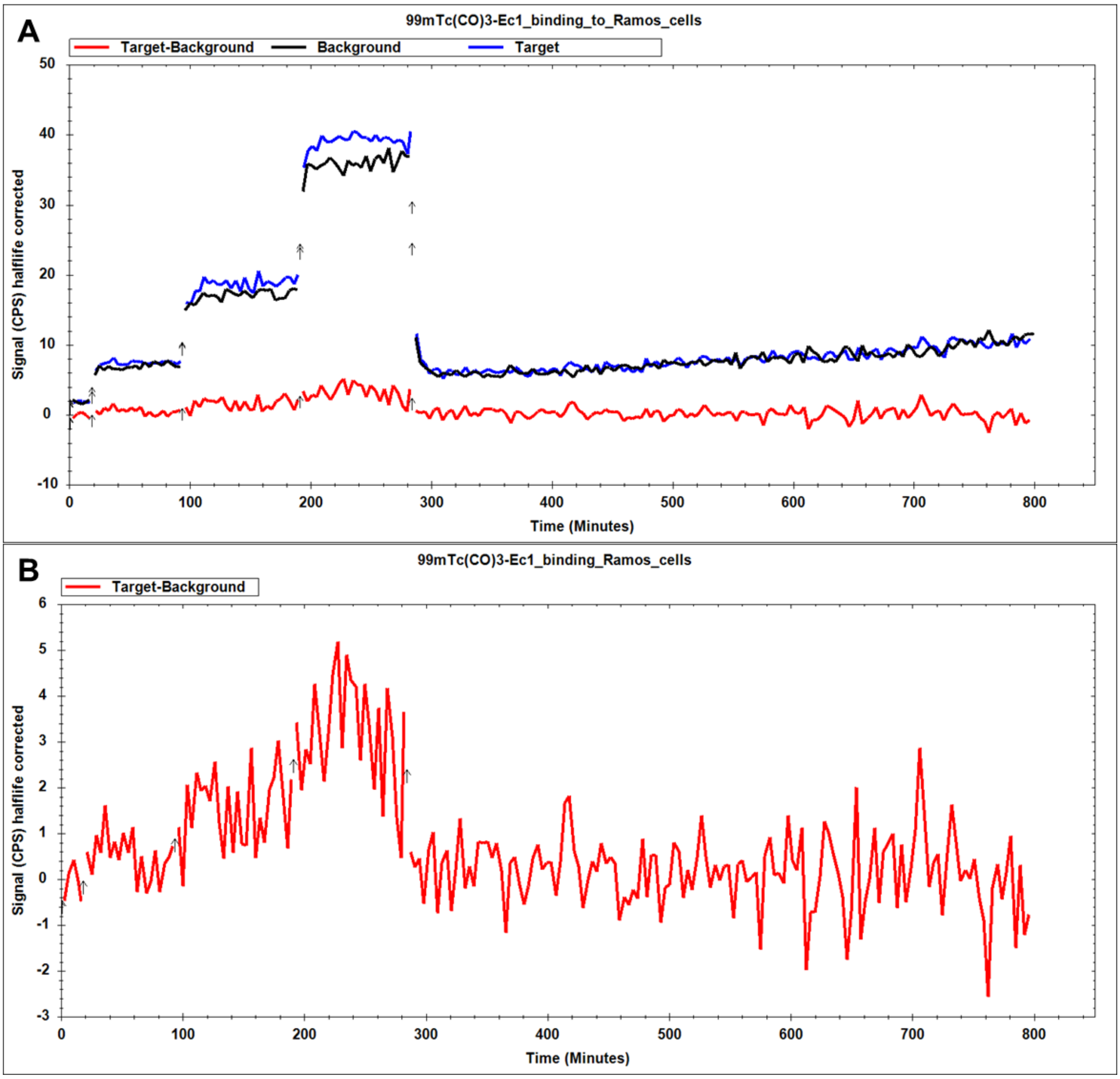

Figure S3. Representative curves of the LigandTracer measurement of [^{99m}Tc]Tc(CO)$_3$-Ec1 binding to Ramos cells. The signal curves display an average of three data points for visibility. (**A**) Signal from the cell-free reference area (plastic area of a Petri dish) is shown in black (Background). Signal from the area with cells is shown in blue (Target). Subtraction of the Background signal from Target signal is shown in red (Target-Background). (**B**) The signal corrected for background (Target-Background, red). Specific activity was 1.6 MBq/µg (start of experiment). The association was measured at 0.2, 0.6 and 1.8 nM concentrations.

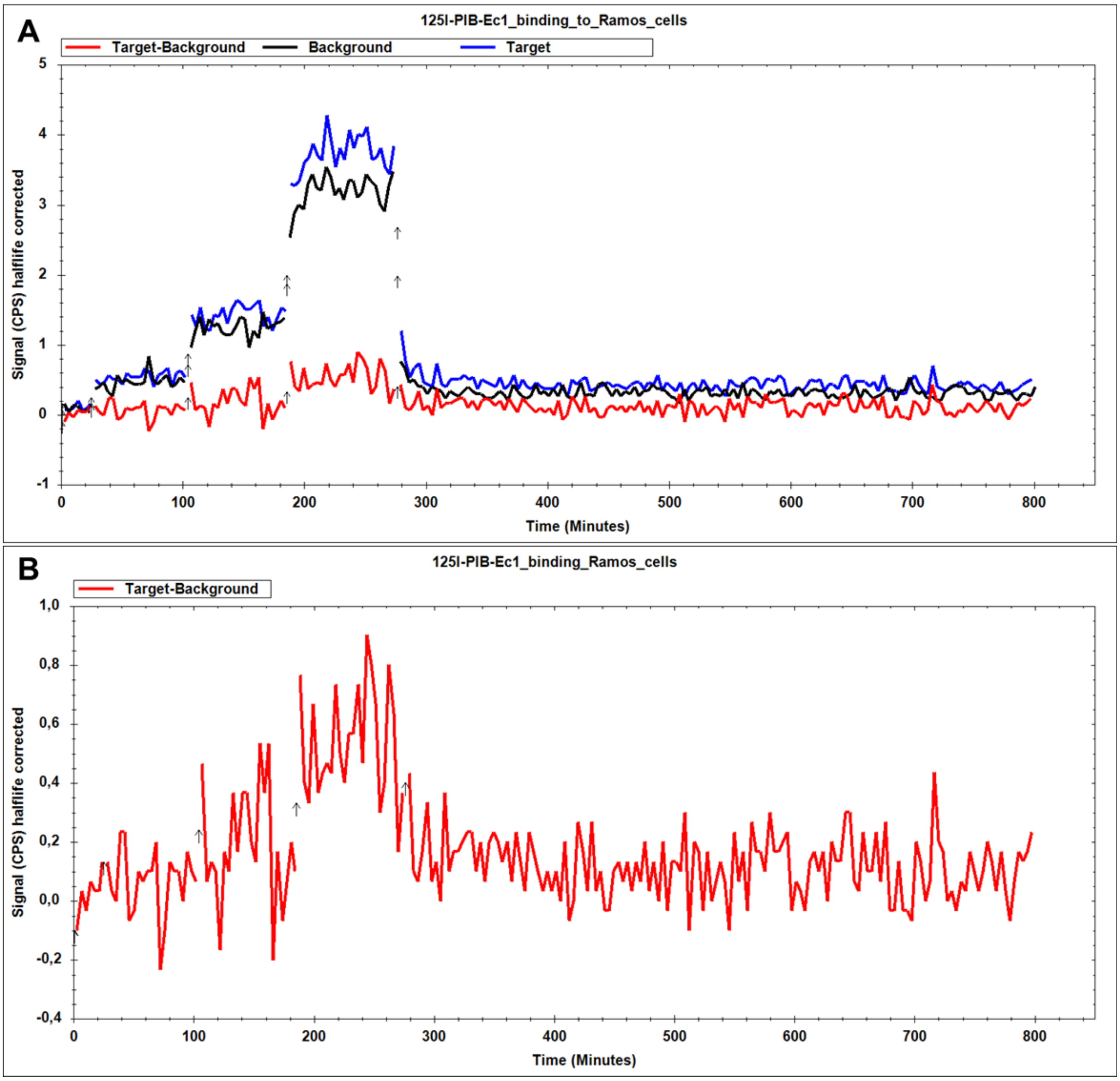

Figure S4. Representative curves of the LigandTracer measurement of [^{125}I]I-PIB-Ec1 binding to Ramos cells. The signal curves display an average of three data points for visibility. (**A**) Signal from the cell-free reference area (plastic area of a Petri dish) is shown in black (Background). Signal from the area with cells is shown in blue (Target). Subtraction of the Background signal from Target signal is shown in red (Target-Background). (**B**) The signal corrected for background (Target-Background, red). Specific activity was 0.02 MBq/µg. The association was measured at 1.8, 5.4 and 14.5 nM concentrations.

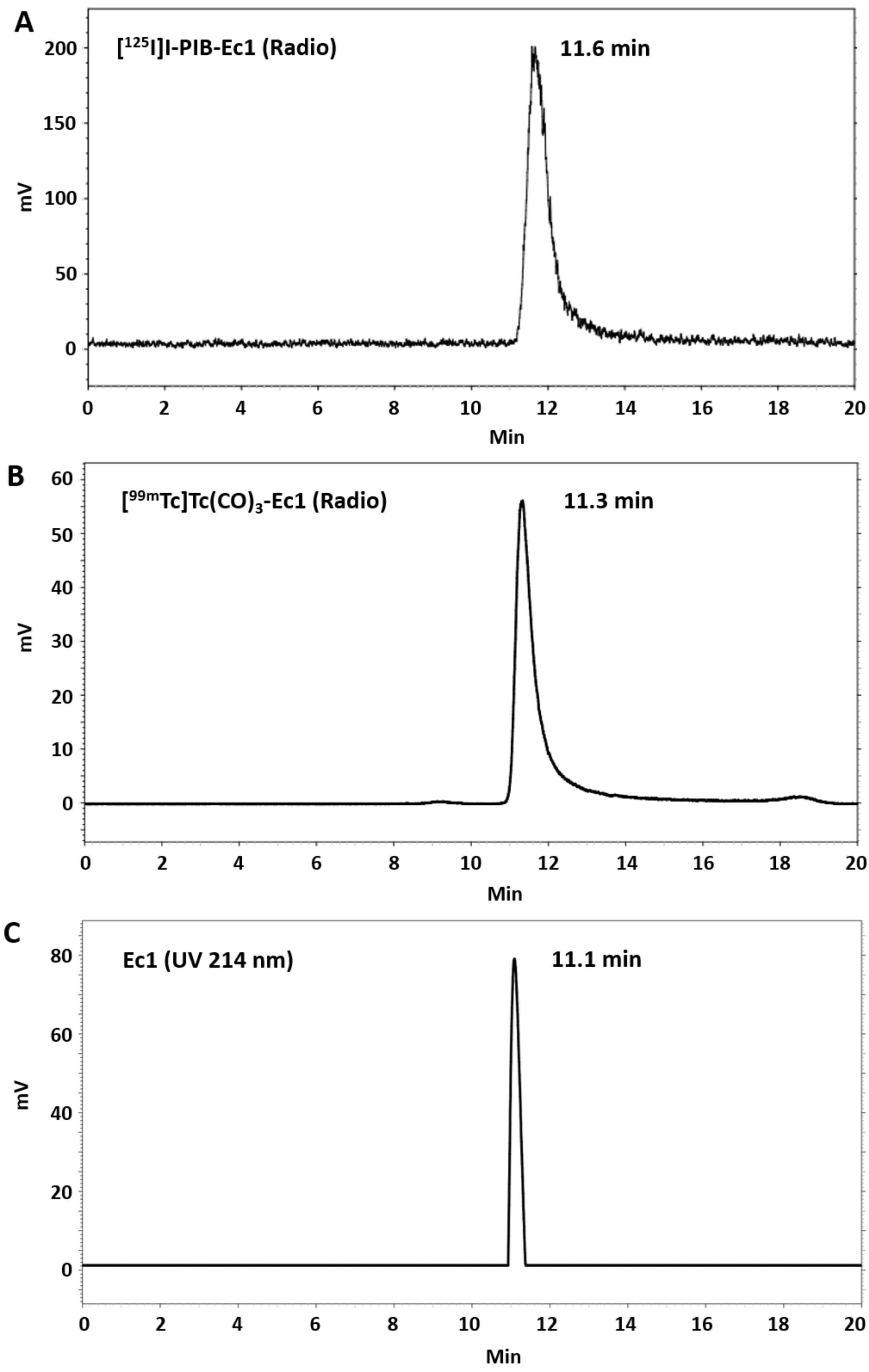

Figure S5. Radio-HPLC analysis of $[^{125}I]$I-PIB-Ec1 (**A**) and $[^{99m}Tc]Tc(CO)_3$-Ec1 (**B**) in comparison to the non-labeled Ec1 (UV at 214 nm).

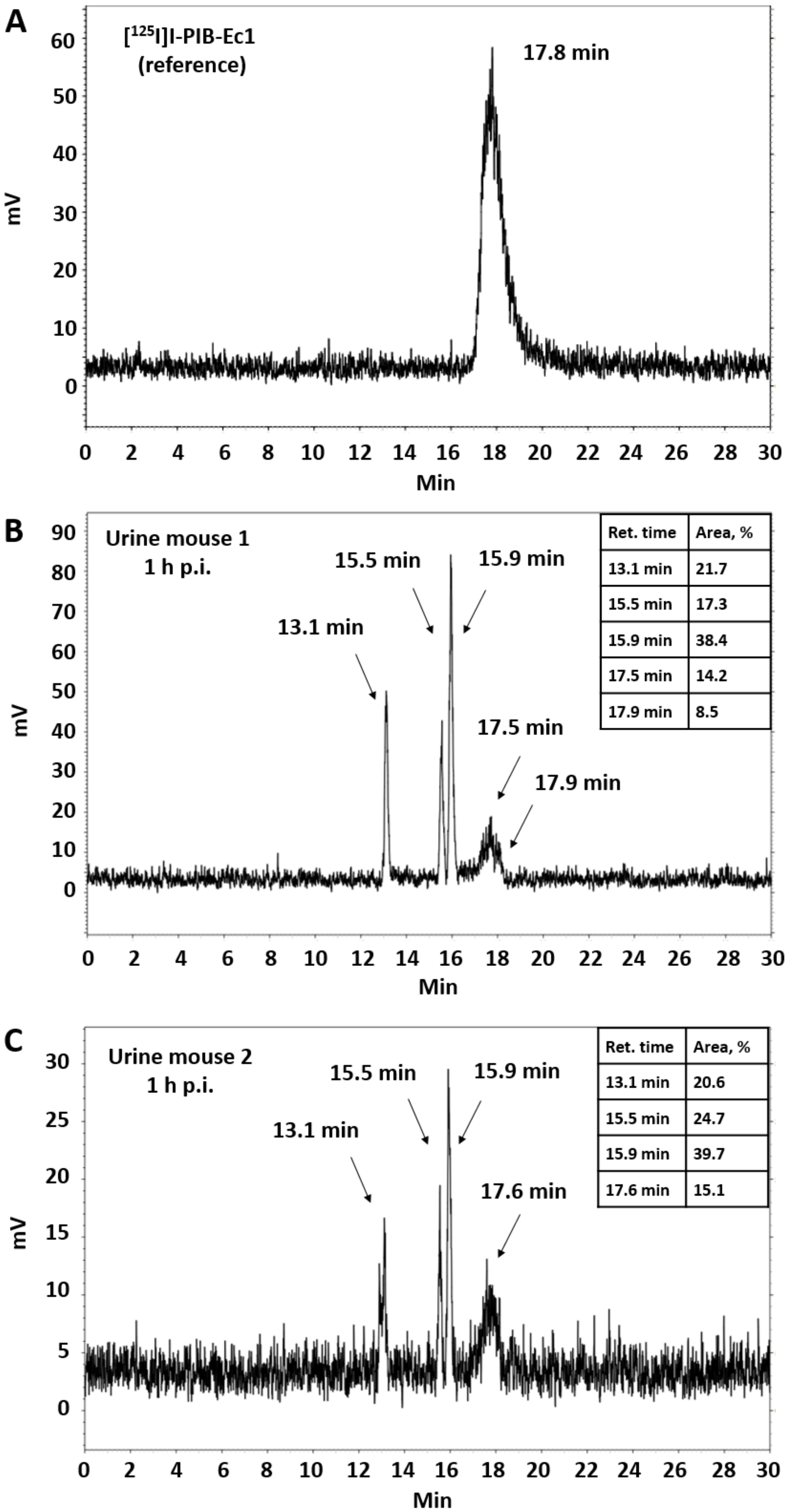

Figure S6. Radio-HPLC analysis of mouse urine 1 h after injection of [^{125}I]I-PIB-Ec1 (from two healthy NMRI mice, **B** and **C**) in comparison to the intact [^{125}I]I-PIB-Ec1 (**A**).

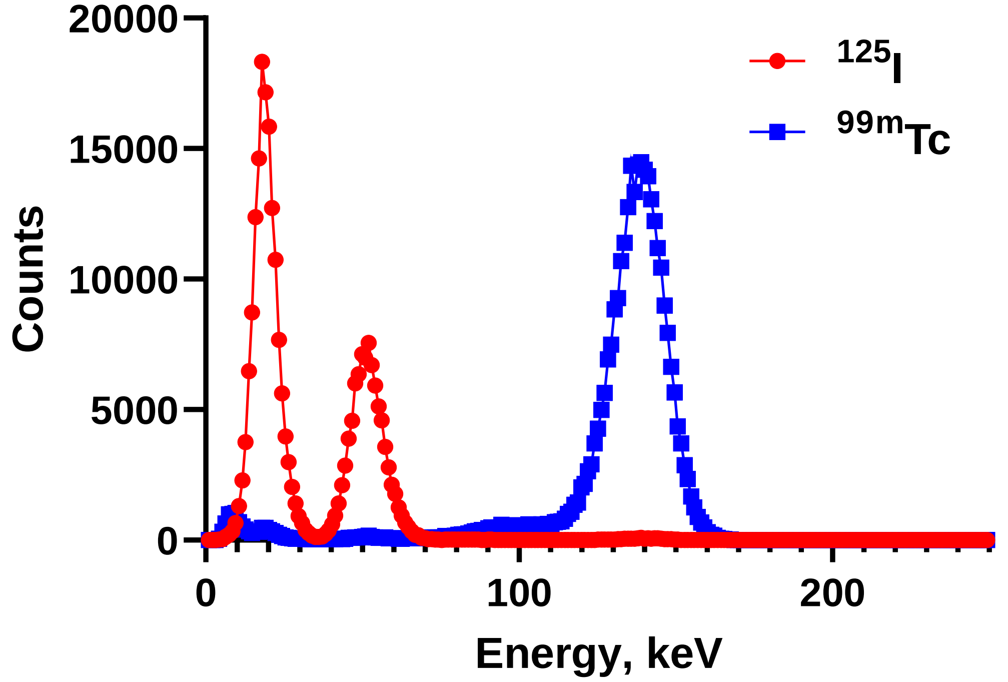

Figure S7. Resolution of gamma-spectra of ^{125}I and ^{99m}Tc. Spectra of authentic samples of ^{125}I and ^{99m}Tc were measured using an automated gamma-spectrometer with a NaI (TI) detector (1480 Wizard, Wallac, Finland).

	**6 h no blocking**		**24 h no blocking**	
**[^{125}I]I-PIB-Ec1**	**Average, %ID**	**SD**	**Average, %ID**	**SD**
blood	0.06	0.02	0.006	0.002
salivary glands	0.01	0.01	NM	NM
lungs	0.027	0.007	0.005	0.001
liver	0.09	0.02	0.029	0.002
spleen	0.009	0.002	0.004	0.001
pancreas	0.006	0.001	NM	NM
small intestine	0.009	0.003	NM	NM
stomach	0.016	0.004	NM	NM
kidney	0.57	0.20	0.018	0.001
tumor	0.12	0.12	0.012	0.002
muscle	0.016	0.007	NM	NM
bone	0.06	0.02	0.05	0.02
intestines	0.6	0.5	0.07	0.01
carcass	1.3	0.1	0.9	0.2
tail	0.5	0.4	0.1	0.0
**SUM**	**3.5**	**0.7**	**1.2**	**0.2**
**[^{99m}Tc]Tc(CO)$_3$-Ec1**	**Average, %ID**	**SD**	**Average, %ID**	**SD**
blood	0.17	0.05	0.08	0.01
salivary glands	0.19	0.09	0.17	0.06
lungs	0.110	0.039	0.075	0.030
liver	14	2	10.0	1.0
spleen	0.29	0.09	0.23	0.04
pancreas	0.17	0.02	0.16	0.09
small intestine	0.13	0.08	0.10	0.04
stomach	0.20	0.05	0.14	0.03
kidney	41	2	27	2
tumor	0.18	0.19	0.07	0.01
muscle	0.20	0.08	0.13	0.05
bone	0.15	0.07	0.13	0.07
intestines	1.8	0.3	1.1	0.1
carcass	11.6	1.2	8.5	1.5
tail	1.4	0.9	0.8	0.1
**SUM**	**72**	**4**	**49**	**1**

Table S1. Biodistribution of [^{99m}Tc]Tc(CO)$_3$-Ec1 and [^{125}I]I-PIB-Ec1 in Balb/c nu/nu mice bearing MDA-MB-468 xenografts (n= 4) at 6 and 24 h. Data are presented as %ID ± SD per whole sample (with rounding using common procedures). NM- not measureable.
